# Dietary Potassium Downregulates Angiotensin-I Converting Enzyme, Renin, and Angiotensin Converting Enzyme 2

**DOI:** 10.3389/fphar.2020.00920

**Published:** 2020-06-18

**Authors:** Carlos P. Vio, Pedro Gallardo, Carlos Cespedes, Daniela Salas, Jessica Diaz-Elizondo, Natalia Mendez

**Affiliations:** ^1^Center for Aging and Regeneration CARE UC, Department of Physiology, Facultad de Ciencias Biológicas, Pontificia Universidad Católica de Chile, Santiago, Chile; ^2^Facultad de Medicina y Ciencia, Universidad San Sebastian, Santiago, Chile; ^3^Facultad de Medicina, Escuela de Medicina, Universidad Finis Terrae, Santiago, Chile; ^4^Facultad de Medicina, Institute of Anatomy, Histology and Pathology, Universidad Austral de Chile, Valdivia, Chile

**Keywords:** dietary potassium intake, renin, angiotensin converting enzyme 2 (ACE2), immunohistochemistry, angiotensin-I converting enzyme (ACE)

## Abstract

**Background:**

The importance of dietary potassium in health and disease has been underestimated compared with that placed on dietary sodium. Larger effort has been made on reduction of sodium intake and less on the adequate dietary potassium intake, although natural food contains much more potassium than sodium. The benefits of a potassium-rich diet are known, however, the mechanism by which it exerts its preventive action, remains to be elucidated. With the hypothesis that dietary potassium reduces renal vasoconstrictor components of the renin-angiotensin system in the long-term, we studied the effect of high potassium diet on angiotensin-I converting enzyme, renin, and angiotensin converting enzyme 2.

**Methods:**

Sprague Dawley male rats on a normal sodium diet received normal potassium (0.9%, NK) or high potassium diet (3%, HK) for 4 weeks. Urine was collected in metabolic cages for electrolytes and urinary volume measurement. Renal tissue was used to analyze angiotensin-I converting enzyme, renin, and angiotensin converting enzyme 2 expression. Protein abundance analysis was done by Western blot; gene expression by mRNA levels by RT-qPCR. Renal distribution of angiotensin-I converting enzyme and renin was done by immunohistochemistry and morphometric analysis in coded samples.

**Results:**

High potassium diet (4 weeks) reduced the levels of renin, angiotensin-I converting enzyme, and angiotensin converting enzyme 2. Angiotensin-I converting enzyme was located in the brush border of proximal tubules and with HK diet decreased the immunostaining intensity (*P* < 0.05), decreased the mRNA (*P* < 0.01) and the protein levels (*P* < 0.01). Renin localization was restricted to granular cells of the afferent arteriole and HK diet decreased the number of renin positive cells (*P* < 0.01) and renin mRNA levels (*P* < 0.01). High potassium intake decreased angiotensin converting enzyme 2 gene expression and protein levels (*P* < 0.01).

No morphological abnormalities were observed in renal tissue during high potassium diet.

The reduced expression of angiotensin-I converting enzyme, renin, and angiotensin converting enzyme 2 during potassium supplementation suggest that high dietary potassium intake could modulate these vasoactive enzymes and this effects can contribute to the preventive and antihypertensive effect of potassium.

## Introduction

Sodium and potassium ions are important for life. Body sodium content maintains extracellular volume and plasma sodium concentration determines plasma osmolality (Slotki and Skorecki, 2016). Body potassium content resides mainly in the intracellular space and a small fraction in the extracellular space. The ratio of intracellular to extracellular potassium concentration determines the membrane potential, which is critical for maintaining normal excitability in nerve and cardiac muscle (Blaustein et al., 2012). Cardiovascular and kidney disease are major causes of mortality worldwide. The kidney is involved in the pathophysiology of arterial hypertension, which in turn is a major risk factor in cardiovascular diseases. High chronic daily sodium intake is a major factor determining endothelial dysfunction and arterial hypertension.

Several epidemiological and meta-analysis studies demonstrate that high dietary sodium intake is associated with high risk of cardiovascular disease including high systolic and diastolic blood pressure and many other cardiovascular and kidney disease (Aaron and Sanders, 2013). High dietary potassium intake reduced systolic and diastolic blood pressure (Tobian, 1997). The reduction of blood pressure associated with high dietary potassium intake was observed only in hypertensive population (Aaron and Sanders, 2013; Mente et al., 2014). Furthermore, it was demonstrated that in hypertensive people, the increase in potassium intake had no adverse effects (Agurto et al., 2013).

The mechanisms responsible for the beneficial effects of a high dietary potassium on the cardiovascular system are incompletely understood. Epidemiological studies demonstrate that there is a negative correlation between potassium excretion and systolic pressure; the relationship is steeper in hypertensive people (Mente et al., 2014). These results suggest that at least the beneficial effect of a high potassium diet on systolic blood pressure is related to the kidneys through renal potassium excretion. Potassium secretion by distal convoluted tubule 2, connecting and principal cells of the distal nephron is the major process involved in potassium excretion (McDonough and Youn, 2017). On the other hand, the distal nephron is also involved in the regulation of extracellular volume and blood pressure through the fine tuning of sodium reabsorption and, at the same level, sodium reabsorption and potassium secretion are intimately related (Penton et al., 2015). A high potassium diet also stimulates the renal kallikrein-kinin system. Kallikrein, secreted by the connecting tubule cells, is an enzyme that mediates the synthesis of bradykinin. This peptide mediates vasodilation and natriuresis by binding to B2K receptors, which in turn stimulates NO and prostaglandin synthesis (Vio and Figueroa, 1987). Therefore, the kidneys seem to be an important target through which a high potassium intake lowers arterial blood pressure.

Several vasoactive systems may contribute to arterial hypertension. The systemic renin-angiotensin system increases arterial blood pressure in several ways. Angiotensin II increases total peripheral resistance (Touyz and Schiffrin, 2000); increases NaCl reabsorption (Gamba, 2012) and stimulates aldosterone synthesis, which in turn increases NaCl reabsorption in the aldosterone-sensitive distal nephron (Féraille and Doucet, 2001). The intrarenal renin-angiotensin system may also contribute to hypertension. Renin enzyme can reach the proximal tubule that express angiotensinogen, angiotensin-I converting enzyme (ACE), and AT1 receptors in the apical domain of proximal tubule and distal convoluted tubule producing angiotensin II that contributes to hypertension through the stimulation of NaCl reabsorption in the proximal and distal convoluted tubule (Velez, 2009; Gurley et al., 2011; Li et al., 2018).

Plasma potassium plays an important role in the regulation of intrarenal vasoactive systems. It was demonstrated in rats, that hypokalemia maintained for 12 weeks increases the expression of cortical ACE and endothelin-1. This protocol of hypokalemia also induced renal injury that was most prominent in the cortex. All these findings favor the development of salt sensitivity and hypertension (Suga et al., 2001). On the other hand, salt sensitivity requires the expression of renal ACE as in mouse models lacking ACE where salt sensitivity in response to renal injury was completely absent (Giani et al., 2015). The cellular mechanisms underlying the effects of hypokalemia on ACE expression are unclear. The intrarenal mechanisms by which a high potassium diet reduces arterial blood pressure, increases natriuresis and diuresis, are incompletely understood. Since the intrarenal renin-angiotensin system favors sodium retention, the inhibition of this system could contribute to the potassium induced natriuretic response. With the hypothesis that a high potassium diet could down regulate the components of the intrarenal renin-angiotensin system, the aim of this research was to study the effect of a chronic high potassium diet on the expression of cortical renin, ACE, and angiotensin converting enzyme 2 (ACE2) in rat kidney.

## Methods

### Animals and Experimental Procedures

Adult male Sprague-Dawley rats (180 to 200 g, n = 11 for each group) were housed under controlled temperature (18–20°C) and a 12 h light/dark cycle. All animals received food and water *ad-libitum* and kept at the animal care facilities of the Pontificia Universidad Catolica de Chile. All experiments were carried out following the directions of the “Manual de Normas de Bioseguridad” version 2008, FONDECYT-CONICYT, and the Guide for the Care and Use of Laboratory Animals of the Institute for Laboratory Animal Research of the National Research Council, and approved by the Institutional Ethical Committee (Fondecyt 1130741).

Rats were randomly assigned to a control group (NK, 0.26% sodium and 0.91% potassium in chow, tap water) or a supplemented potassium diet (HK, 0.26% sodium and 2% potassium in chow, 2% KCl in water), with free access to food and water for 4 weeks. During the first week of treatment, the HK group received 1% KCl in drinking water. Then, KCl was raised to 2% from week 2 until the end of the treatment. Normal (Prolab RMH 3000) or potassium supplemented chow (Prolab RMH 3000 supplemented with potassium to a final concentration of 2% potassium) were purchased from Purina LabDiet. The sodium and potassium content were verified at the Laboratory of Food Analysis at the Instituto de Ciencia y Tecnologia de los Alimentos, Universidad Austral de Chile, Valdivia, Chile.

For urine collection, rats were placed in metabolic cages (Tecniplast, Buguggiate, Italy) for 16 h (from 5 pm to 9 am) during the last day of the 4 week period; water and food were supplied *ad libitum*. Urine samples were obtained, measured, and pelleted at 1,000 g for 10 min and stored at −20°C. After the sampling period, rats were anesthetized with isoflurane (isoflurane + O_2_, induction 4–5% and maintenance 1–2%). A blood sample was obtained from Vena Cava in heparin-tubes, both kidneys were removed, isoflurane was increased until breathing stopped and death of the animals was confirmed. Pneumothorax was performed as a secondary physical method of euthanasia.

Kidney capsule was rapidly removed. Whole kidney sections were obtained for immunohistochemistry, Western blot, and qRT-PCR. The samples for Western blot and qRT-PCR were rapidly frozen in liquid nitrogen and stored at −80°C until processing. Serum concentration and urinary excretion of potassium and sodium were determined using an ion selective electrolyte analyzer 9180 (Roche Diagnostic).

### Source of Antisera and Chemicals

Antibodies. Anti-α-tubulin antibody (T-5168) were purchased from Sigma Chemical; anti-ACE antibody (SC-12187), anti-goat IgG-HRP (SC-2020) were from Santa Cruz Biotechnology (Santa Cruz, CA); secondary antibody and corresponding peroxidase-anti-peroxidase (PAP) complex were from MP Biomedicals (Santa Ana, CA). Anti-renin antibody for immunohistochemistry was a gift from Dr. Pierre Corvol (College de France, Paris, France), ACE2 antibody for Western blot was from Abcam (Cambridge, UK).

Reagents. MMLV, DNAse I, TRIzol reagent and phosphatase inhibitor were from Invitrogen (Carlsbad, CA); dNTPs, random primers, FAST SYBR Green Master Mix, from Thermo Fisher Scientific (Waltham, MA). ECL Western blotting substrate was from Pierce (Waltham, MA).

### Tissue Processing and Immunohistochemical Analysis

Renal tissue samples (3-mm thick) were fixed by immersion in Bouin's solution for 24 h at room temperature, dehydrated, embedded in paraffin (Paraplast Plus, Sigma-Aldrich), and serially sliced at 5 μm thickness with a Leica rotary microtome. The samples were mounted on glass slides and stored for immunohistochemistry. Renin and ACE immunostaining was performed using an indirect immunoperoxidase technique in randomly selected kidney samples (Vio et al., 2012; Vio et al., 2018). Briefly, kidney sections were dewaxed, rehydrated, and rinsed in 0.05 M Tris-phosphate-saline (TPS) buffer pH 7.6. The kidney sections were incubated overnight at 22°C with a primary antiserum raised against renin or ACE. After rinsing in TPS buffer, the secondary antibody and corresponding PAP complex were applied for 30 min each at 22°C. The immunoreactive sites were revealed after incubating the sections in 0.1% (wt/vol) diaminobenzidine and 0.03% hydrogen peroxide. The sections were rinsed with TPS buffer, counterstained with hematoxylin, dehydrated, and cleared with xylene. Controls for the immunostaining procedure were prepared by omission of the first antibody. The kidney sections were examined with conventional light microscopy and acquired using a Nikon Eclipse E600 microscope and Nikon DS-Ri1 digital camera. For renin and ACE, the stained area in each image was quantified using Simple PCI, a computer-assisted image-analysis software, Hamamatsu Corporation (Sewickley, PA). The values corresponding to total immunostained area, were averaged and expressed as fold change *versus* control.

### Western Blotting

Western blotting was performed as described previously (Vio et al., 2012; Vio et al., 2018). In brief, kidney sections weighing 100 mg were homogenized in lysis buffer (150 mM NaCl, 50 mM Tris·HCl, 0.5% deoxycholate, 0.1% SDS, NP40) containing protease and phosphatase inhibitors (phosphatase inhibitor cocktail 1 and 2) with an Ultra-Turrax. After centrifugation at 14,000 rpm for 10 min, protein concentration was determined using the BCA method. Equal amount of protein extract (50 μg total) was mixed with sample buffer (100 mM Tris·HCl, pH 6.8, 200 mM dithiotreitol, 4% SDS, 0.2% bromophenol blue, and 20% glycerol) and heated at 75°C for 10 min. The protein samples were separated in 10% Tris-glycine SDS-PAGE and transferred to a PVDF membrane. The membrane was soaked in blocking solution 5% skim milk in TTBS (Tris-buffered saline and Tween 20) for 1 h at room temperature and incubated with a primary antibody against ACE or ACE2 overnight at 4°C. The membranes were washed in TTBS, and incubated with the horseradish peroxidase (HRP)-conjugated secondary antibody for 1 h at room temperature. Immunoreactivity in the membrane was detected with SuperSignal West Dura Extended Duration Substrate (Thermo Fisher Scientific) and stored with ChemiDoc-It Imaging System (UVP, Upland, CA).

### Quantitative RT-PCR

Total RNA was extracted from frozen kidney samples using TRIzol reagent according to the manufacturer instructions. RNA integrity was determined in 1% agarose gel electrophoresis. Total RNA concentration was determined by measuring the absorbance at 260/280 nm. cDNA synthesis was carried out with 2.5 µg of total RNA incubated with MMLV reverse transcriptase, dNTPs, and random primers according to instructions provided by the manufacturer. After incubation, samples were treated with DNAse I. For quantitative PCR, the primer sequences used were the following:

ACE, forward primer: 5'-AACACGGCTCGTGCAGAAG-3',
ACE, reverse primer: 5'-CCTGCTGTGGTTCCAGGTACA-3';
ACE2, forward primer: 5'-ACCCTTCTTACATCAGCCCTACTG-3',
ACE2, reverse primer 5'-TGTCCAAAACCTACCCCACTAT-3';
Renin, forward primer: 5'-GCTACATGGAGAATGGGACTGAA-3',
Renin, reverse primer: 5'-ACCACATCTTGGCTGAGGAAAC-3'.

Quantitative PCR was performed in duplicate in a StepOnePlus Real-Time PCR System (Applied Biosystems) using FAST SYBR Green Master Mix for amplification. Results were normalized by glyceraldehyde 3 phosphate dehydrogenase (GAPDH) forward primer: 5'-CACGGCAAGTTCAACGGC-3' reverse primer 5'-GGTGGTGAAGACGCCAGTA-3'. Mathematical quantification was carried out using the 2^-ΔΔCT^ method (Livak and Schmittgen, 2001).

### Statistics

All data are presented as mean ± SEM. Statistical analyses were performed using an unpaired Student's t-test and GraphPad Prism software (La Jolla, CA). Differences with P < 0.05 were considered statistically signiﬁcant.

## Results

The effect of a chronic high potassium diet (HK) on serum potassium and sodium concentration, and urinary excretion of sodium and potassium are shown in **Table 1**. As expected, plasma sodium concentration did not change between both groups and plasma potassium concentration was slightly increased in the HK diet group compared to control group (3.80 ± 0.08 mEq/L *versus* 4.46 ± 0.26 mEq/L; P < 0.05). The effect of a high potassium diet on sodium excretion is shown in **Table 1**. Compared to control rats, the HK group increased significantly its urinary sodium (0.92 ± 0.08 *versus* 1.87 ± 0.31; P < 0.01) and urinary potassium (3.60 ± 0.23 *versus* 30.30 ± 4.32; P < 0.01) excretion.

**Table 1:** Serum and urinary electrolytes.

Group (diet)	Serum concentration (mEq/L)		Urinary excretion (mEq/16 h)	
	Na^+^	K^+^	Na^+^	K^+^
**NK**	**140.80 ± 1.07**	**3.80 ± 0.08**	**0.92 ± 0.08**	**3.60 ± 0.23**
**HK**	**139.80 ± 0.66**	4.46 ± 0.26*	1.87 ± 0.31**	30.30 ± 4.32**

Effect of a chronic high potassium diet on serum electrolytes and urinary excretion in adult male rats. The potassium supplementation diet (HK) did not alter significantly the serum Na^+^ concentration. Serum K^+^ concentration was increased significantly. In the HK group, urinary sodium excretion was doubled compared to control diet. Urinary potassium excretion was also significantly increased. Data are expressed as mean ± SEM; n = 11; *P < 0.05, **P < 0.01.

In this study immunohistochemical staining was used to characterize the protein expression at tissue level and the distribution of the vasoactive enzymes ACE and renin in the kidney.

Renal tissue samples were examined in coded fashion by two of us (C.Cespedes and C.Vio), and systematic analysis of kidney tissue samples was done focusing on the overall morphological aspect, and on cortical and medullary tubules, glomeruli, blood vessels, interstitial space, and intratubular spaces as previously done (Vio et al., 2018).

**Figure 1:**
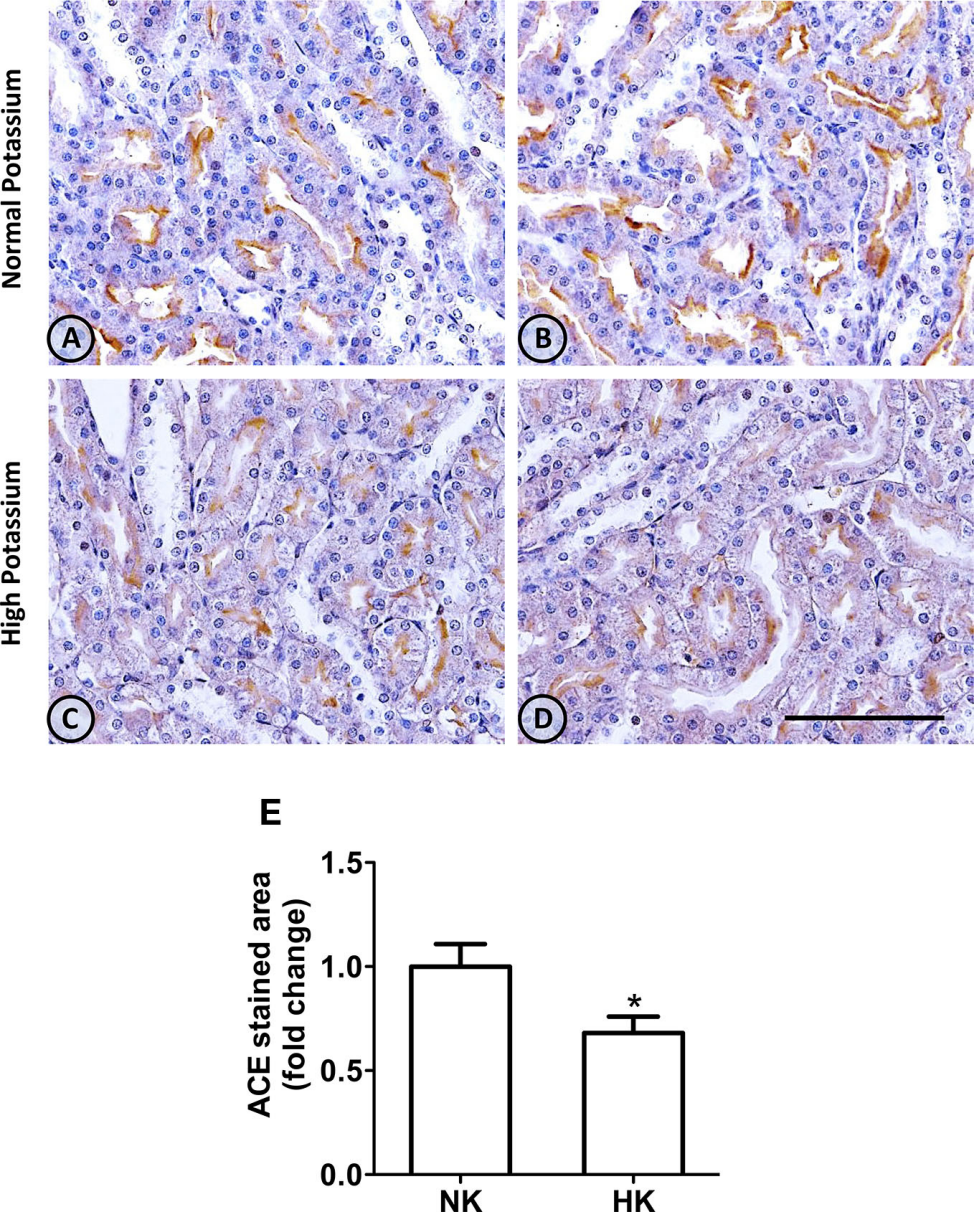
ACE immunolocalization in normal (NK) and high potassium (HK) diet. **(A, B)** Light micrographs of kidneys from different rats on normal potassium diet group. ACE immunoperoxidase signal is restricted to the apical domain of proximal straight tubules. **(C, D)** Light micrographs from kidneys of rats on the high potassium diet show the reduction in ACE immunoperoxidase signal. **(E)** Morphometric analysis of the stained area in micrographs of kidneys of both groups. The high potassium diet decreased significantly the immunostained area compared to control diet (mean ± SEM; n = 11; *P < 0.05). Scale bar for all micrographs = 100 µm.

An overall and detailed examination of renal tissue with immunostaining and hematoxylin of kidneys from animals from both groups showed no signs of pathological alterations in the cortex or the medulla. No vascular changes were observed, tubules were normally shaped in terms of diameter and cell size, and no signs of cell infiltration or inflammation were observed in the tubulointerstitial space. Glomeruli from cortical or juxtamedullary nephrons had a normal aspect (**Figures 1** and **4**).

### Vasoactive Enzyme Expression and Distribution

#### Angiotensin-I Converting Enzyme

**Figure 2:**
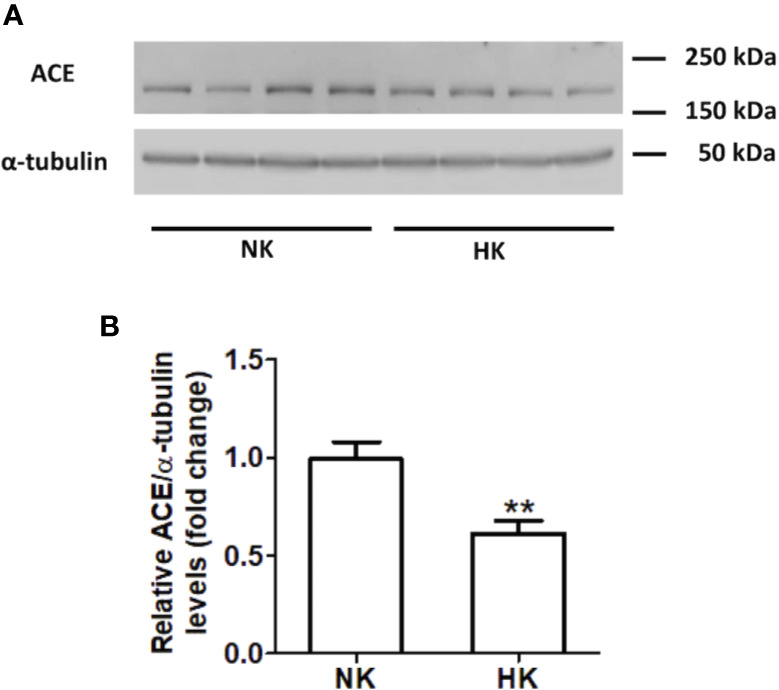
ACE protein abundance in normal (NK) and high potassium diet (HK). **(A)** Immunoblot for ACE using total renal protein; **(B)** densitometric analysis of ACE immunoblot shows a significant reduction in ACE protein abundance in the high potassium diet group compared to control group (mean ± SEM; n = 11; **P < 0.01).

**Figure 3:**
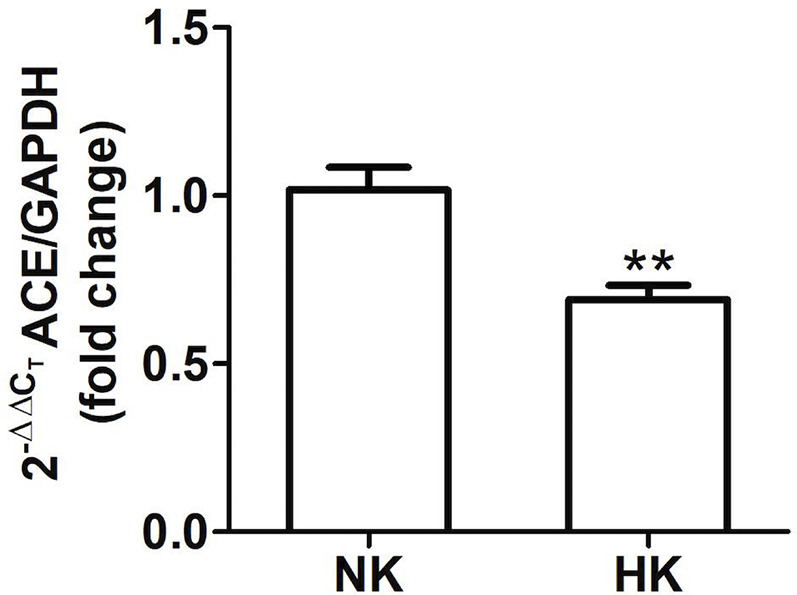
Quantitative real time PCR for ACE mRNA in control (NK) and high potassium (HK) diet group. Potassium supplementation in the diet markedly reduces the mRNA abundance of ACE compared to the control group (mean ± SEM; n = 11; **P < 0.01).

The immunolocalization of ACE is shown in **Figure 1**. In the kidneys from control rats (**Figures 1A, B**), ACE was localized in proximal straight tubules. The immunoperoxidase signal was mainly present in the apical domain; scarce or none immunostaining was seen in the cytoplasm and no staining was observed at the basolateral domain, as reported previously (Vio and Jeanneret, 2003). The immunoperoxidase signal was markedly reduced in the high potassium diet group (**Figures 1C, D**) compared to the control diet group. In the HK group, localization of ACE immunostaining remained in the proximal straight tubules. The subcellular localization of the immunoperoxidase signal was also conserved compared to control group. However, the morphometric analysis of the ACE immunostained area (**Figure 1E**), showed a significant decrease in the HK group compared to control group. The reduction of the ACE immunoperoxidase signal found in the experimental group suggest a reduction in ACE protein abundance. To investigate if the reduction in ACE immunostaining observed in the HK group was accompanied by a reduction of protein abundance, we performed Western blot analysis with total kidney protein (**Figure 2A**). The densitometric analysis of bands (**Figure 2B**) showed that ACE protein abundance was significantly reduced in the HK group. Quantitative RT-PCR was used to determine if the high potassium diet induced changes of ACE at mRNA level. As shown in **Figure 3**, the ACE mRNA abundance is markedly reduced in the HK group. Therefore, the high potassium diet supplementation produced significant reductions in ACE mRNA and protein abundance, which could also be observed as a reduction in ACE immunoperoxidase signal in the proximal tubules.

#### Renin

**Figure 4:**
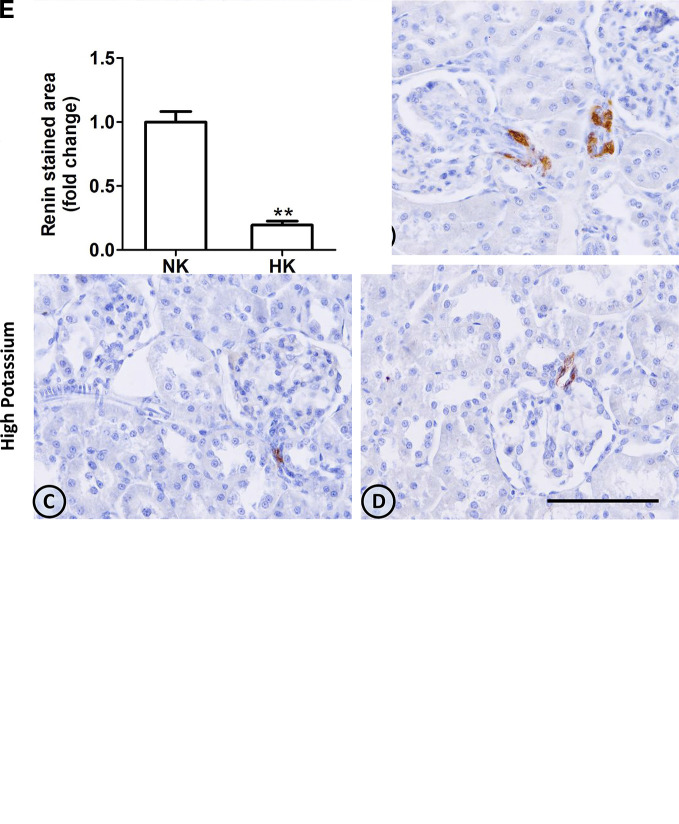
Renin immunolocalization in control (NK) and high potassium (HK) diet. **(A, B)** Light micrographs of two different normal kidneys showing renin immunoperoxidase signal only in afferent arterioles. **(C, D)** Images from different kidneys of rats in a high potassium diet. Renin immunolocalization is conserved, but the number of positive immunostained cells is reduced. **(E)** Morphometric analysis of the immunostained area for renin shows a marked decrease in the high potassium diet group (mean ± SEM; n = 11; **P < 0.01). Scale bar for all micrographs = 100 µm.

**Figure 5:**
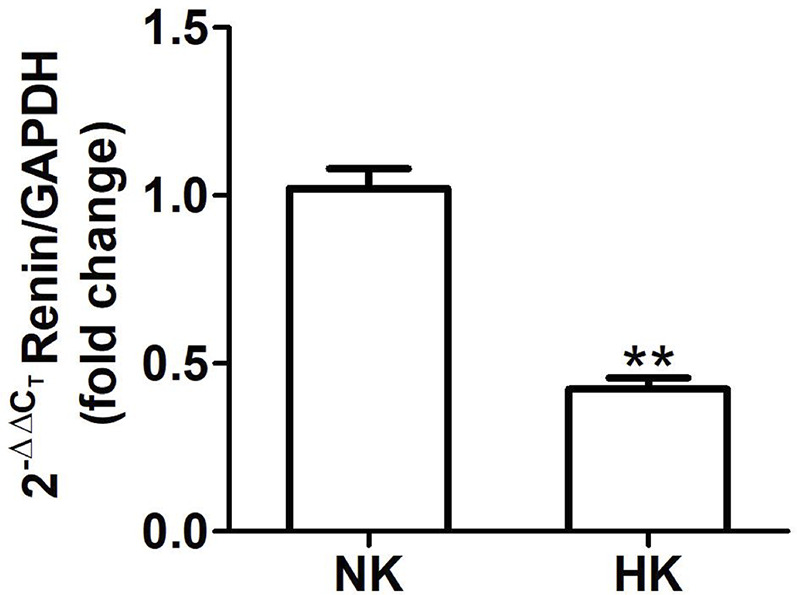
Renin mRNA abundance in control (NK) and high potassium (HK) diet. Renin mRNA abundance is reduced in rats with chronic high potassium diet compared to control rats (mean ± SEM; n = 11; **P < 0.01).

The effect of HK diet on renin localization was investigated through immunohistochemistry. The renin immunoperoxidase signal was detected only in renal cortex. Specific localization of renin immunoperoxidase signal was restricted, in both groups, to juxtaglomerular cells located in the afferent arterioles (**Figure 4**). The HK diet reduced the number of juxtaglomerular cells positive for renin in the wall of afferent arterioles, as evidenced by the significant reduction in the renin stained areas showed in the morphometric analysis (**Figure 4E**). The HK diet did not modify the localization of the renin immunoperoxidase signal. Quantitative RT-PCR was performed to determine if the HK diet affected renin mRNA abundance. As shown in **Figure 5**, a significant reduction in renin mRNA abundance was found in the HK group. We performed Western blot analysis to determine if the HK diet affected renin protein abundance. However, our results did not clarify whether renin protein abundance was reduced by a high potassium diet. Therefore, our results show that a high potassium diet reduced renin mRNA level. This finding could explain the reduction of the renin immunoperoxidase signal observed in afferent arterioles of animals fed a HK diet.

#### Angiotensin Converting Enzyme 2

**Figure 6:**
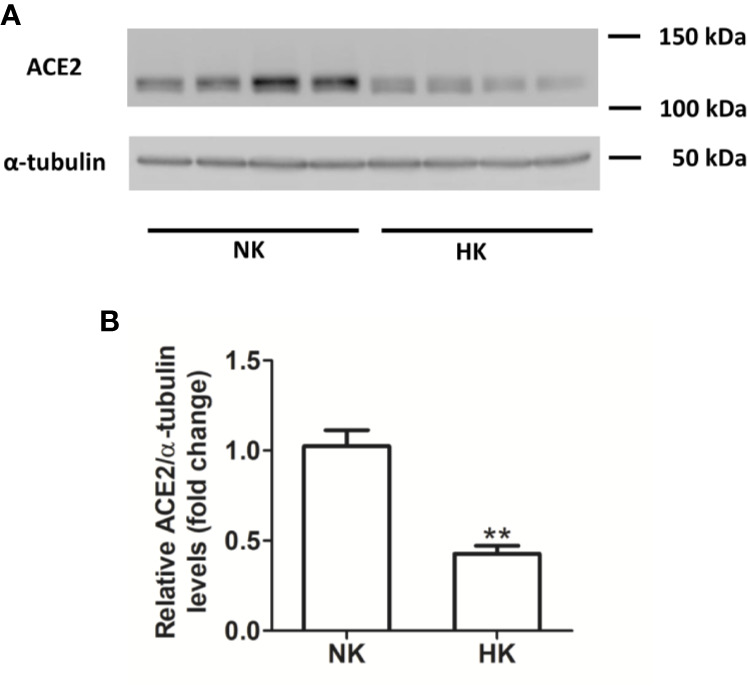
ACE2 protein abundance in normal (NK) and high potassium diet (HK) **(A)**: ACE2 immunoblot using total renal proteins of control (NK) and high potassium (HK) diet. **(B)**: Densitometry of bands show a significant decrease in ACE2 protein abundance in the HK group (mean ± SEM; n = 11; **P < 0.01).

**Figure 7:**
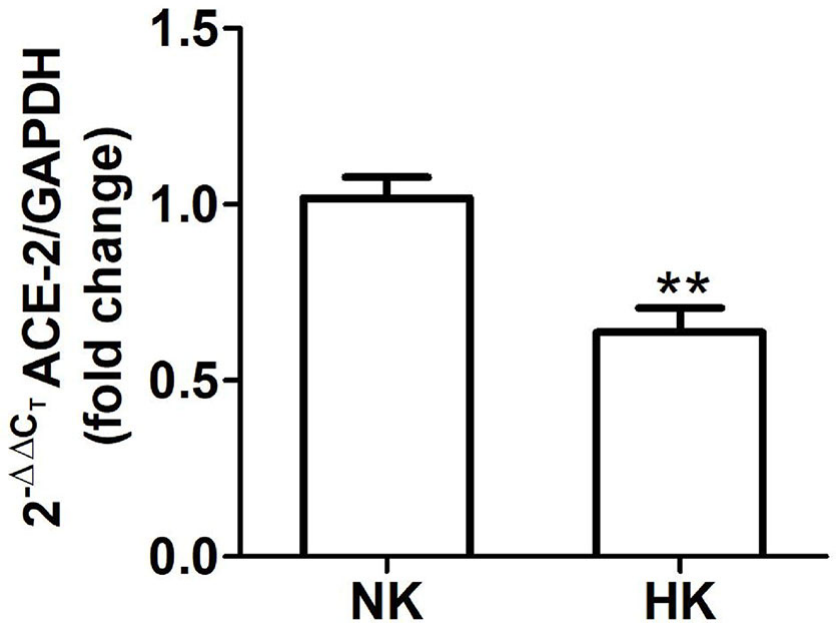
ACE2 mRNA abundance in normal (NK) and high potassium (HK) diet. ACE2 mRNA abundance is reduced in the high potassium group (mean ± SEM; n = 11; **P < 0.01).

We also investigated the effect of HK diet on ACE homolog ACE2 at the protein and mRNA level. The effect of the chronically HK diet on ACE2 protein abundance was investigated through Western blot analysis (**Figures 6A, B**), showing that ACE2 protein abundance was markedly reduced. Quantitative RT-PCR also revealed a significant reduction in ACE2 mRNA abundance (**Figure 7**). In contrast with the results obtained with ACE and renin, no positive immunostaining for ACE2 was observed in renal structures, despite the use of several commercial antibodies.

## Discussion

There is a large body of evidence showing that potassium supplementation reduces blood pressure, but the mechanisms are still not fully understood. The main findings of the present study are that chronic potassium supplementation reduces the abundance of mRNA and protein of ACE and ACE2 and also reduces the number of renin-positive cells and its mRNA, with no signs of pathologic lesions in the kidney.

The experiment to demonstrate the effect of a high potassium diet was set for 4 weeks. During the first week the rat were fed with 2% potassium in chow plus 1% KCl in drinking water and increased to 2% KCl by the second week. Control rats received 0.91% potassium in chow and tap water. Measurements of electrolyte urinary excretion as well as plasma Na^+^ and K^+^ were done in the last day of the treatment. Therefore, during the 4-week period, the kidneys of the HK group undergone potassium adaptation. This phenomenon allowed a higher urinary potassium excretion to compensate a higher potassium ingestion and the maintenance of potassium balance. Physiological and morphometric studies carried out in control and potassium loaded rats for 4 weeks demonstrated that chronic renal potassium adaptation and high urinary potassium excretion is supported by increased potassium secretion and basolateral membrane area in connecting and principal cells of the distal nephron (Stanton et al., 1981). Although not evaluated directly, we can assume that our HK group is in steady state in relation to potassium since an increased ingestion was accompanied with an increased urinary excretion.

As previously stated, all measurements were carried out under steady state rather than acute conditions. The high potassium diet produced an increase in natriuresis and potassium urinary excretion. These results are consistent with other chronic experiment, where rats were fed for 8 days with a high potassium diet and K^+^-induced natriuresis was also doubled (van der Lubbe et al., 2013). Potassium-induced natriuresis was also demonstrated in acute experiments. In mice receiving an oral KCl load through a gastric gavage, K^+^-induced natriuresis increased 30–60 min after the administration (Sørensen et al., 2013).

The functioning of the high potassium diet induced natriuresis depends on coordinated action of several mechanisms. The increase in dietary K^+^ produces a small increase in plasma K^+^ concentration that is sensed by Kir 4.1/5.1 potassium channel at the basolateral membrane (Su et al., 2019; Wu et al., 2019). The potassium channel inhibition closes a basolateral chloride channel. The increase in intracellular chloride concentration inhibits the WNK/SPAK/OSRI signal cascade and the activation of apical NCC (Terker et al., 2015). This latter inhibition increases NaCl delivery to the connecting and cortical collecting tubule.

It has been stated that that the preceding mechanism is not enough to enhance K^+^ secretion in connecting cells and principal cells. Kir 4.1 is expressed in the basolateral membrane of connecting and principal cells (Sørensen et al., 2019). An increase in plasma K^+^ inhibits the channel and increases intracellular K^+^ concentration. Recently it was demonstrated that cell K^+^ activates mTORC2, which in turn phosphorylates SGK1. Activated SGK1 inhibits ENaC retrieval from apical membrane, this increasing the driving force for K^+^ secretion. This novel local mechanism stimulates K^+^ secretion necessary to maintain K^+^ homeostasis in the presence of a high dietary potassium.

Angiotensin-I converting enzyme plays a key role in intrarenal vasoactive peptide systems. The enzyme transforms angiotensin I into angiotensin II inducing vasoconstriction and also inactivates bradykinin, a vasodilator peptide (Li et al., 2018). In our experiments, ACE immunolocalization was mainly present in the brush border of epithelial cells of proximal straight tubules although ACE had been localized also in mesangial cells and collecting duct (Redublo Quinto et al., 2007). In our experiments, chronic potassium supplementation did not modify the subcellular localization of the enzyme but ACE immunostaining and protein abundance were reduced. The reduction in protein abundance was also accompanied by a decrease in ACE mRNA levels. Therefore, the potassium supplementation reduced both ACE mRNA and protein abundance. As a consequence, there should be a reduction in the conversion rate of angiotensin I into its active peptide angiotensin II. This peptide is a very important stimulator of proximal tubule Na^+^ reabsorption by enhancing apical NHE3 exchanger activity (Geibel et al., 1990). Thus, less formation of intratubular angiotensin II could explain the increased natriuresis induced by high potassium in addition to the described dephosphorylation of the renal sodium chloride cotransporter in response to oral potassium intake (Sørensen et al., 2013; Penton et al., 2015).

The mechanisms responsible for the decrease in ACE mRNA and protein are incompletely understood, but could be attributed to a decrease in the transcription rate, mRNA stability, or an increase in mRNA degradation. There is little information regarding ACE regulation. Studies carried out in human cardiac fibroblast showed that dexamethasone increased ACE abundance by transcriptional and post-transcriptional mechanisms (Barretto-Chaves et al., 2001). In endothelial cells, it was demonstrated that ACE expression is under the control of β-adrenergic stimulation. The effect was reproduced by Gs protein stimulation, adenylyl cyclase stimulation, cAMP analogs and was blocked by propranolol and PKA inhibitors (Xavier-Neto et al., 1999). Our results cannot elucidate if one of these mechanisms is operating under our experimental conditions. Further experiments need to be done in order to understand the mechanism behind ACE mRNA and protein reduction induced by high potassium diet.

In contrast to our observation of ACE reduction with a high potassium diet, low potassium diet increases ACE expression (Suga et al., 2001). Given the importance of ACE in the generation of angiotensin II (the main pro-hypertensive peptide), it seems logical that both low and high potassium regulate its abundance. It remains to be elucidated whether ACE reduction and overexpression share the same molecular mechanisms.

Juxtaglomerular cells are the source of cortical renin in the normal kidney (Castrop et al., 2010). In both normal and high potassium diet groups, renin immunolocalization was restricted to cells at the glomerular end of the afferent arterioles. However, in the high potassium group the number of renin positive cells in afferent arterioles was reduced. The morphometric analysis showed a reduction of the renin immunostaining in the afferent arterioles area in the high potassium group. In the same group, the reduction in renin immunoperoxidase signal was accompanied by a significant decrease in renin mRNA levels. The reduction in renin positive cells in immunohistochemistry studies and the reduction of mRNA abundance in high potassium group suggest a reduction in the renin protein abundance. It has been shown that a high salt diet decreases cortical renin and its abundance (Castrop et al., 2010). The reduction of renin found in the high potassium diet group could not be attributed to high sodium intake, since in both groups the amount of sodium in pellet was the same. We could not demonstrate, through Western blot, if the chronic high potassium diet affects renin protein abundance. Problems with Western blots to identify cortical renin seem a common technical issue and might explain the scarcity of published cortical renin Western blots in the literature.

Even though we did not measure plasma renin activity, the reduction of plasma renin activity has been demonstrated in acute and chronic potassium loading protocols. In an acute K^+^-loading protocol carried out in dogs, Vander demonstrated a reduction of plasma renin activity in venous blood of dogs that received a KCl infusion through the renal artery (Vander, 1970). Chronic experiments carried out in rats that received a high potassium diet for 8 days, also showed a reduction in plasma renin activity (van der Lubbe et al., 2013). Therefore, either an acute or chronic potassium loading results in a reduction of plasma renin activity. Since renin can be filtered in the glomerular barrier (Li et al., 2018), a decrease in filtered renin implies a decrease in formation of angiotensin I from angiotensinogen, which can be secreted by proximal straight tubules (Navar et al., 2011). Therefore, a reduction in the number of renin positive cells could be related to the observed reduction in plasma renin activity in animal models of potassium loading.

As stated above, the reduction in ACE and renin expression induced by high potassium diet could imply a reduced rate of intratubular angiotensin II formation, which in turn is a key stimulator of NHE3 exchanger in the proximal tubule brush border (Geibel et al., 1990). Since NHE3 mediates one third of proximal Na^+^ reabsorption, a reduction in its activity or abundance might be one of the mechanisms involved in potassium-induced natriuresis. Experiments carried out in male and female mice submitted to a 7 day period of a high potassium diet showed a gender-independent significant reduction in NHE3 protein abundance in the high potassium group. This effect could not be explained by changes in NHE3 gene expression since no change was observed in the NHE3 mRNA abundance in high potassium and control group (Yang et al., 2018). The mechanism responsible for NHE3 protein down regulation in the high potassium group remains unknown.

The apical sodium chloride cotransporter (NCC) cotransporter of the distal tubule is another target of angiotensin II through a WNK4-SPAK dependent pathway. SPAK-dependent NCC phosphorylation increases cotransporter activity in the apical membrane (Gamba, 2012). Thus, a reduced formation of angiotensin II could account for the marked reduction in NCC phosphorylation that was reported in mice after an acute oral potassium load (Sørensen et al., 2013). In chronic potassium loading experiments, total NCC and phosphorylated NCC protein abundance were drastically reduced in the high potassium group (Yang et al., 2018). Therefore, the reported reduction of protein abundance of proximal tubule NHE3 and distal convoluted NCC could explain the increased natriuresis found in the high potassium group in our experiments.

As stated above, ACE also mediates bradykinin degradation to inactive peptides, therefore a reduction in ACE could lead to an increase of intrarenal bradykinin (Rhaleb et al., 2011). Furthermore, it is recognized that a high potassium diet increases kallikrein synthesis and secretion in the connecting cells (Vio and Figueroa, 1987). Thus, a high potassium diet generates a setting in which ACE is reduced and kallikrein is increased. Both conditions favor intrarenal bradykinin formation. Recent studies demonstrate that the activation of B2 bradykinin receptors in the distal tubule results in the inhibition of NCC and Kir4.1 mediated potassium conductance (Zhang et al., 2018). Taken together, the phenomenon of potassium-induced natriuresis could be explained not only by the reduction of the activity of the intratubular renin-angiotensin system but also by the upregulation of the kallikrein-kinin system.

ACE2 is an ACE homolog expressed in the kidney and many other organs (Soler et al., 2013; Santos et al., 2018). ACE2 mRNA expression was studied in microdissected nephron segments; the highest abundance was found in proximal straight tubule (Li et al., 2005). Western blot and immunohistochemical studies revealed that ACE2 protein has a wide tubular and vascular distribution in the renal tissue. However, the immunohistochemistry did not clarify the exact subcellular localization of ACE2 protein. Mass spectrometry studies carried out with isolated proximal straight tubules from rat kidney suggest that ACE2 converts angiotensin I to angiotensin 1-7 and angiotensin 1-9; the latter is converted to angiotensin 1-7 by ACE (Li et al., 2005).

In our study, ACE2 mRNA abundance was reduced in kidney from rats exposed to high potassium diet. Consistently, the same group exhibited a reduction in ACE2 protein abundance. Unfortunately, none of the several primary antibodies tested were useful to visualize the cellular localization and subcellular distribution of ACE2. Therefore, we could not demonstrate any possible effect of the chronic high potassium diet on ACE2 tissue distribution and subcellular localization. There are few studies with the localization of ACE2 in the kidney (proximal tubules and podocytes) and they are in mice kidneys (Ye et al., 2006), perhaps species differences could explain our lack of visualization of ACE2 in rat kidneys. The effect of the high potassium diet on ACE2 mRNA and protein abundance could imply a reduction in intrarenal angiotensin 1-7 formation. Since the proximal straight tubule has the highest ACE2 expression, it is possible that a high potassium diet could reduce angiotensin 1-7 formation in this tubular segment. It has been shown that intrarenal ACE2 expression and activity has beneficial effects that could be eliminated in the HK group due to the reduction of intrarenal ACE2. However, the histology of renal cortex of HK group is preserved compared to control group. On the other hand, studies of arterial pressure under the same diet protocol revealed that rats were normal (Gonzalez et al., 2019).

The effects of angiotensin 1-7 on tubular NaCl and water reabsorption are conflicting. Micropuncture studies carried out in rat kidney showed a lack of effect of physiological concentrations of intratubular angiotensin 1-7 on proximal tubule fluid reabsorption, loop of Henle and distal tubule (Vallon et al., 1997). In other study carried out in rat isolated microperfused proximal straight tubules, 1 pM angiotensin 1-7, added to the bath, stimulated fluid and bicarbonate reabsorption. This stimulatory effect was reduced at higher concentration of the peptide in the bath (Garcia and Garvin, 1994). Since the effect of angiotensin 1-7 on tubular NaCl and water reabsorption in unclear, it is very difficult to envision a mechanism involving angiotensin 1-7 in the potassium induced natriuretic effect.

In summary, our experiments demonstrate that a chronic high potassium diet down regulates key components of intratubular renin-angiotensin system. This down regulation could be a novel mechanism involved in the potassium induced natriuresis that is beneficial for blood pressure and cardiovascular health. Further experiments should be carried out to determine the mechanisms that mediate the reduction in the expression of the intrarenal renin-angiotensin system.

## Data Availability Statement

The datasets generated for this study are available on request to the corresponding author.

## Ethics Statement

The animal study was reviewed and approved by Comite Etico Cientifico of PontificiaUniversidad Catolica of Chile.

## Author Contributions

CV, DS, and CC designed the study. DS performed gene expression. JD-E performed protein expression. CC performed immunohistochemical studies. CC and NM were responsible for carrying experiment in animals. PG, DS, CC, and JD-E analyzed data and made the figures. PG and CV analyzed data, drafted and revised the paper. All authors contributed to the article and approved the submitted version.

## Funding

This research was funded by Grants PIA CONICYT AFB170005 to CARE-UC and Fondecyt 11170245 to NM, and a donation of SQM to the Pontificia Universidad Catolica de Chile.

## Conflict of Interest

The authors declare that the research was conducted in the absence of any commercial or financial relationships that could be construed as a potential conflict of interest.
